# Supplementary material for: Ontogenetic shape trajectory of Trichomycterus areolatus varies in response to water velocity environment
Source: PLoS One. 2021 Jun 11;16(6):e0252780. doi: 10.1371/journal.pone.0252780 (PMC8195363; doi:10.1371/journal.pone.0252780)
Supplement: Attachment — Submitted filename: Searle et al PLOS ONE_R2.pdf [file pone.0252780.s002.pdf]

Review: Ontogenetic shape trajectory of Trichomycterus areolatus varies in response to stream gradient

Re-Review:

The revision by Searle et al. is improved from the first submission, but the writing needs clarification throughout the manuscript. I have detailed line-by-line locations in the manuscript where the authors can consider clarifying, bolstering or specifying their arguments.

I still have a major issue with the take-home message being that stream gradient is the major feature driving the ontogenetic trajectories between the two groups. This is because your metric for quantifying stream gradient is the degree of river slope, which is 0.14 in the low-flow and 0.4/0.2 in the "high flow." I understand that you grouped transitional flow with high so that you could have sufficient datasets for comparison, but I am not convinced this was the right way to go. Why combine transition flow data with high flow when the river slope in the transition is 0.2 degrees? This is closer to 0.14 of the low flow. Can you provide other data for flow in the low vs. high regimes, such as flow rate or flow volume? Your argument is that stream gradient is driving different ontogenetic trajectories in this species of fish. This is a super cool take-home message, so it needs to be clear that this is indeed the case.

The second major issue is in the interpretation or importance/novelty of the findings that ontogenetic trajectory is mediated by environment. The statistics indicate that this is the case (through the interaction), but there is no further clarity or understanding of what this means in terms of HOW ontogeny is redirected in a low- vs. high-flow environment. What happens to the shape of these fish during development? I understand that high-flow are more fusiform with longer heads, but you don't need ontogenetic data to show this. What is new from this study is that you can detail what specific shape changes occur throughout ontogeny between the two environments. You might consider bolstering this argument.

I totally understand that it is deflating to receive a less-than-laudatory review. Your paper is getting there, but it needs (1) clarity in the writing, (2) further interpretation on how environment mediates ontogenetic trajectory (this is the novelty of your dataset) and (3) to reconcile the stream gradient issue (especially if this is the take-home message, i.e., in the title of your paper).

General comment not touched on below:

- Wider heads and larger eyes in high flow could also have to do with prey type in high-flow environments. Prey type is a potential source of selection that could produce similar results.

Line-specific comments:

27 – Sentence remains confusing (and it is important that this sentence is clear). Are you saying that head shape and body shape vary with respect to one another? This is kind of how this sentence reads.

46 – This paragraph is on intraspecific variation in morphology, while the previous is on interspecific variation? I think you could clarify this for the reader. Also, you might include what the selective pressures were to cause these intraspecific morphological divergences in each example you provide.

50 – Finally, for this paragraph, you might be careful with your wording in the last sentence. By suggesting that phenotypic variation can result from either natural selection or phenotypic plasticity, you are implying that plasticity is not under the control of natural selection, which I would argue is not true. Perhaps what you mean to say is that variation can have a genetic or plastic component? If this is true, you should clarify this language.

52 – Your paragraph on ontogenetic shape change does not do justice to the vast literature on ontogenetic allometry among fishes, let alone vertebrates. From On Growth and Form (1917) to present day there are thousands of studies on the ontogeny of form. It would behoove you to give this subject more treatment both in the introduction and discussion of your paper.

74 – "select for" or "result in/produce?" Select for implies a specific understanding of adaptive significance behind phenotypic change. In this case you are saying that environment would select for a particular ontogenetic strategy (i.e., fast growth rate or something). I think what you are trying to say is that environment can mediate ontogenetic trajectory to produce a different adult morphology from a similar juvenile starting morphology?

74 – 78 – It is unclear what is happening to male/female morphology between high and low predation environments. Are males the same throughout development or do they start different and undergo parallel shape change? Females start different but end up the same (converge)? This need clarification.

78 – 80 – Are you saying here that in this stickleback study the three environments produced three different morphologies? Or that marine differed from fresh? Please clarify. Also, is it that these studies did not look at shape change throughout development and that your study is doing this? It would be good for you to clarify exactly what has been done and where your study fits in.

80 – 82 – Consider deleting "in different environments over ontogeny" – this part of the sentence is repetitive.

107 – Are you saying here that you grouped the environments into high-flow and low-flow and acknowledge that there are other features of these environments that might be causing the observed shape change that you quantify (other than flow)? You ultimately suggest (in the title)

that shape change is a product of the stream gradient (which definitely implies flow), and not the confounding variables that also vary with environment.

I remain unconvinced of your reasoning to group the transitional zone with the high-flow. Your whole argument is in how flow speed shapes morphology, but the only values you present on flow speed are the slopes of these different zones (0.4, 0.2 and 0.14 degrees). You group the 0.4 and 0.2 together (lines 120-123) and compare this to the 0.14 and find difference in morphology and this is great, but I don't know what to make of those differences in a functional way as a result of the "two different environments." Can you provide a metric of flow velocity among the three sites, or flow volume (cubic feet per second) to better distinguish low- vs. high-flow?

131 & 134 – Were these two totally different data sets for body shape and head shape? Or did you use mostly the same specimens, just one less for the head shape and 10 more for body shape? Also, were individuals tracked to compare head and body shape of the same individual? This would be important in the statistical analysis (i.e., repeated measures).

298 – I think I know what you are trying to say with this opening sentence, but it is awkward. It reads as if ontogeny is a selective pressure. Same with life history and environment. I believe that environment can provide selection, but I'm not sure that ontogeny and life histories are selective pressures so much as they are traits that can adapt as a result of selection. You should consider opening this section with a strong statement of your major result.

306 – You might consider being more specific here with what your results can say about how environment shapes ontogeny. You do not discern between genetic basis (that these are distinct populations that have evolved different developmental programs) and plasticity (that these populations are responding to their environment). It would take another study to tease these effects apart, but it could be worth acknowledging or specifying here what your data are capable of saying.

308 – What is your metric for fusiform shape? How was this calculated? The only other mention of "fusiform" is in the introduction section so it is hard for the reader to determine how you calculated this variable.

312 – Consider a new paragraph when transitioning to juveniles. This is a massive paragraph otherwise.

321 – Is "endorheic" the correct term here (closed drainage basin)? Does your study river terminate into a lake/basin? For those unfamiliar this might be worth clarifying. Also, you are talking about high flow environments in the preceding sentence, so a clearer transition is needed.

327 – Negative allometry in eye size (relatively large eyes in juveniles) is observed in most ontogenetic studies of fish and may be a developmental constraint more than a product of adaptation.

332 – 335 – Please clarify what you are saying here. Is the high flow environment punctuated by open basins of little-to-no flow? Are you saying that the benthic substrate of these environments is different? Please be more specific and clarify your point.

342 – 346 – This is the first mention that there are no reproductive barriers to gene flow within your study river system. This should come much earlier, or if you do mention it earlier, should be emphasized, because it is important to your findings, specifically that morphological differences might not have a distinct genetic basis and therefore would be the result of phenotypic plasticity.

<u>Original Review:</u>

The study by Searle et al. uses morphometrics to investigate ontogenetic shape change in *T. areolatus* between two different flow environments. The authors find that ontogeny has the greatest effect on morphological shape change, followed by flow regime. The authors report that across environments, *T. areolatus* **become more uniformly-shaped along the body and wider in the head with increasing size**. Secondly, the authors find that between environments, **high flow is associated with narrower bodies and heads laterally**, but **wider heads and larger eyes and shorter/rounder noses**. The interpretation is that this is the acquisition of a more fusiform body shape in high flow regimes and is the result of phenotypic plasticity in response to environmental flow regime.

This is a nice, clean and simple study that is worthy of publication, but the writing and general interpretations need work. Be cautious with your interpretations – external landmarks are difficult to repeatedly place at homologous structures. Be sure to define your landmarks explicitly in the methods. Also, I wonder how much of the overall pattern is being driven by ontogeny alone. Could you consider comparing the adults alone to see if flow regime has an effect? Otherwise, it is tough to convince the readers that these populations differ with respect to flow regime.

Minor comments:

27 – among fishes?

49 –Do you mean developed or evolved?

50-52 – This is a tough transition. Consider the main topics in this paragraph and how they flow from one to another. Is this second paragraph about how fishes adapt to differing flow rates or is it about intraspecific adaptation?

53-56 – Do you need to define natural selection and phenotypic plasticity here? If so, consider your definition of natural selection.

91-94 – Can you provide more convincing reasoning behind grouping the transitional zone with the high flow? The gradient looks closer to the low flow. Generally, this seems problematic if you are trying to say something about how flow influences adaptive morphologies.

106-111 – Perhaps you can be more specific in terms of the breakdown of sizes? Bin them or tell the readers about the distribution? Reporting average size when you intentionally collected over a broad size range only tells the readers that the two groups have the same average size but nothing about the distribution of sizes.

111 – Can you provide details to the level that the method can be replicated? The resolution of photos makes a difference in the precision/accuracy of your morphometric analysis. Camera and lens make/model, etc.

122 – Is this the proper term for the rostral-most point on T. areolatus? Is the point at the tip of the premaxilla? Might try to be more specific here.

123 –Can you be more specific with Point 5 in terms of the importance of this point, why you placed it here and what it tells the reader?

135 – Can you be more specific about point 2? Is this the occipital crest? Remember that these points must be homologous for proper landmarking.

136-137 – It is unclear what point 6 means and how it is repeatedly placed. Is this the lateral-most boundary of the head at the level of the eye (i.e., the widest part of the head)? Perhaps you could say “lateral extent of the head indicated by the intersection of a line drawn perpendicular to points 1 & 2 that passes through the eye and the lateral aspect of the head along that line.” Or something more explicit.

162 – “12 rows (body) or 9 (head) rows” Consider being consistent about where parentheticals are placed in this sentence. It is helpful to the reader.

187-188 – Your explanation of Index methodology (above) is helpful in understanding how the interaction was developed. However, the way the topic sentence here is written is confusing/misleading. You state body shape differed significantly by flow and centroid size – but that is not what you report. I suggest you be explicit in how you open up the major finding of your results by stating exactly what was different, which is that body shape differs based on centroid size and between the three-way interaction of flow, centroid and index – this is different than saying that body shape differed sig by flow – which is what the reader takes away from this topic sentence, but is not necessarily true.

193-194 – Is ontogeny driving the larger patterns you are seeing, particularly with your index interaction? How can you tease apart the effect of flow within this? I'm afraid that the significance of your index and all interactions using that term are driven by the overarching effect of centroid size. Clarifying how you can separate out these effects would be helpful to readers.

198 – You report the magnitude of shape change is significantly different between flow regimes. Be sure to cite the statistical results supporting this comment.
210-211 – Same comment as line 187-188. The way you word this sentence is confusing and possibly misleading.

240 – The reporting of eye size is not obvious within Results. Is this true that adults have relatively larger eyes? I don't see that in the warps and it doesn't make sense with the allometry of eye size among other fishes. Eye size is strongly negatively allometric across ontogeny among fishes. Please check this reporting in your manuscript. Perhaps high flow adults have relatively larger eyes than low flow adults, but the way it is worded here suggests that eye size scales with positive allometry among high flow populations, and this should be clarified and expanded upon, if true.

241-242 – Be sure to include the proper statistics in reference to this comment. Additionally, does this mean that high flow fish retain juvenile features into adulthood? Could you comment on this from a heterochrony/paedomorphic standpoint? Perhaps this is how the environments are mediating the ontogenetic trajectory of these fish – by maintaining juvenile-like characteristics among the high flow populations.

250-251 – It looks like the dorsal view of the head for warp 2 shows a wider aspect of the head at the mouth. I do not disagree that this could still be adaptive for the high flow environment, but I would argue that it shows a more fusiform shape. You will need to be more explicit here and in the results on what the shape changes between environments really are.

259 – Check this report of adults with relatively smaller eyes with what you report previously.

265-267 – There is no citation for the comment on apparent lack of reproductive barriers to gene flow and isolation of populations. Is this personal observation?

269 – You might consider stating at the end of the sentence something along the lines that a common garden experiment would have to be performed to directly address this inference.

Figure 3 – It is difficult to see much difference among the warp line drawings. Consider a figure where they are made larger for the reader to see what is going on. Also, it is tough to interpret the data points on the graph. The data points are confined to as small section of the total space of the plot. Finally, low flow and high flow fish fell along perfectly flat, parallel lines? Perhaps zooming in could give the reader better resolution of the data points.
