# Supplementary material for: Ontogenetic shape trajectory of Trichomycterus areolatus varies in response to water velocity environment
Source: PLoS One. 2021 Jun 11;16(6):e0252780. doi: 10.1371/journal.pone.0252780 (PMC8195363; doi:10.1371/journal.pone.0252780)
Supplement: Attachment — Submitted filename: Response to Reviewers.docx [file pone.0252780.s001.docx]

I have now received two reviews. Both of them are as you can see fairly positive to the manuscript. However, both reviewers also pointed out that the manuscript needs substantial improvement, especially in the overall writing but there are also parts that need more explanation and justification. So, the success of the manuscript critically depends on how you deal with the reviewer's comments in the review process.

In particular, you need to justify the selection procedure of relative warps in the statistical methods (e.g. lines 160-163, see the comments of reviewer 2).

We have justified the use of a subset of relative warps as those that accounted for more than 1% of total shape variation, and we have cited papers that use this same selection criterion. See lines 180-182.

For example, it is not clear what morphological axis a significant interaction explains.

To be clear, our analysis is a multivariate mixed model, such that all selected relative warps are used simultaneously in the analysis. Thus, the interactions are based on the summed effect across multiple relative warps. To give an indication of how relative warps contributed to differences among groups, we included a new figure that plots least squares means for each relative warp for each size by flow environment combination. See lines 213-216 and Figures 3 and 5. We now explain in the results section how each relative warp is related to the overall shape change documented by the significant interaction terms. See lines 244-247 and 273-275.

There are also other parts in the description of the statistical methods that are confusing pointed out by both reviewers. Also for example lines 170-174 make little sense.

We have now revised this sentence to indicate that we are testing for shape change across all relative warps simultaneously. See lines 205-209.

You also need to show more convincing evidence to whether the overall morphology pattern is driven by flow regimes or ontogeny alone, which is the major point of the article (see the comments of reviewer 1). This is especially important since currently it is not clear how you tease these two effects apart.

To be clear, the significant three-way interactions from our multivariate mixed model analysis (for both head and body) indicate that it is not just ontogeny or flow environment, but an interaction between the two. To make this clearer to the reader, we have added two new analyses to our paper. First, we have conducted a phenotypic change vector analysis that specifically tests for differences in magnitude and direction of multivariate shape change between flow environments over ontogeny. This analysis shows that in the body view ontogenetic shape change vectors differ significantly between low and high flow environments in magnitude, but not in direction. In the head view, ontogenetic shape change vectors differ significantly between low and high flow environments in direction, but not in magnitude. See lines 217-228 for an explanation of the methods of analysis and see lines 247-250 and 275-279 for the results of the analysis. The second analysis is a calculation of a divergence vector that allows us to visualize the maximum divergence due to flow environment at small and large sizes. We provide new thin-plate spline visualizations of these divergence patterns for comparison among groups. See figures 4 and 6 and lines 229-238 for an explanation of the methods of analysis and see lines 250-254 and 279-283 and for the results of the analysis. With the help of these two new analyses, we think the nature of this interesting interaction is made clear.

Both reviewers have also given a number of comments and suggestions and when you submit your revised manuscript you need to explicitly state, point by point in how you have dealt with the reviewers’ comments.

In your Methods section, please provide additional information regarding the permits you obtained for the work. Please ensure you have included the full name of the authority that approved the field site access and, if no permits were required, a brief statement explaining why.

We have included a complete explanation of how collections were made and what guidelines we used. These collections were made in 2003 and 2004 and at that time in Chile this sampling was completed under the supervision of the Dirección de Investigación, Universidad de Concepción and the Undersecretariat of Fisheries with no formal permit being required. We have clearly stated the guidelines we used in lines 110-115.

3.In your Data Availability statement, you have not specified where the minimal data set underlying the results described in your manuscript can be found. PLOS defines a study's minimal data set as the underlying data used to reach the conclusions drawn in the manuscript and any additional data required to replicate the reported study findings in their entirety. All PLOS journals require that the minimal data set be made fully available. For more information about our data policy, please see <http://journals.plos.org/plosone/s/data-availability>.

The data set used for analysis has been uploaded to Dryad <https://datadryad.org/stash/share/lC_kykyvRxHgybaB10KANtwGOfBNtgf888BdqBmqDxw>

(Private link). The public DOI will be provided upon formal acceptance of the manuscript.

**Comments to the Author**

Review Comments to the Author

Reviewer #1: Review: Ontogenetic shape trajectory of Trichomycterus areolatus varies in response to stream gradient

The study by Searle et al. uses morphometrics to investigate ontogenetic shape change in T. areolatus between two different flow environments. The authors find that ontogeny has the greatest effect on morphological shape change, followed by flow regime. The authors report that across environments, T. areolatus become more uniformly-shaped along the body and wider in the head with increasing size. Secondly, the authors find that between environments, high flow is associated with narrower bodies and heads laterally, but wider heads and larger eyes and shorter/rounder noses. The interpretation is that this is the acquisition of a more fusiform body shape in high flow regimes and is the result of phenotypic plasticity in response to environmental flow regime.

This is a nice study that is worthy of publication, but the writing and general interpretations need work. Be cautious with your interpretations – external landmarks are difficult to repeatedly place at homologous structures. Be sure to define your landmarks explicitly in the methods.

We have added clarifying text to tell how we reduced the amount of noise generated by inaccurate placement of landmarks. See lines 144-151. Briefly, we 1) carefully reviewed each photograph before landmarking, 2) had one researcher landmark specimens randomly without reference to collection location or body size, and 3) had two other researchers quality check the landmarks placed by one individual to confirm homologous placement of points.

Also, I wonder how much of the overall pattern is being driven by ontogeny alone. Could you consider comparing the adults alone to see if flow regime has an effect? Otherwise, it is tough to convince the readers that these populations differ with respect to flow regime.

To be clear, the significant three-way interactions from our multivariate mixed model analysis (for both head and body) indicate that it is not just ontogeny or flow environment, but an interaction between the two. To make this clearer to the reader, we have added two new analyses to our paper. First, we have conducted a phenotypic change vector analysis that specifically tests for differences in magnitude and direction of multivariate shape change between flow environments over ontogeny. This analysis shows that in the body view ontogenetic shape change vectors differ significantly between low and high flow environments in magnitude, but not in direction. In the head view, ontogenetic shape change vectors differ significantly between low and high flow environments in direction, but not in magnitude. See lines 217-228 for an explanation of the methods of analysis and see lines 247-250 and 275-279 for the results of the analysis. The second analysis is a calculation of a divergence vector that allows us to visualize the maximum divergence due to flow environment at small and large sizes. We provide new thin-plate spline visualizations of these divergence patterns for comparison among groups. See figures 4 and 6 and lines 229-238 for an explanation of the methods of analysis and see lines 250-254 and 279-283 and for the results of the analysis. With the help of these two new analyses, we think the nature of this interesting interaction is made clear.

Minor comments:

27 – among fishes?

Agreed. We changed “in fish” to “among fishes.” See line 27.

49 –Do you mean developed or evolved?

The introduction has been significantly revised and we no longer use this phrase in the text.

50-52 – This is a tough transition. Consider the main topics in this paragraph and how they flow from one to another. Is this second paragraph about how fishes adapt to differing flow rates or is it about intraspecific adaptation?

We have rewritten much of the introduction to provide better flow among paragraphs. See lines 46-56.

53-56 – Do you need to define natural selection and phenotypic plasticity here? If so, consider your definition of natural selection.

For readers of Plos, we agree that the definitions for natural selection and phenotypic plasticity are not needed. They have been removed from the manuscript.

91-94 – Can you provide more convincing reasoning behind grouping the transitional zone with the high flow? The gradient looks closer to the low flow. Generally, this seems problematic if you are trying to say something about how flow influences adaptive morphologies.

We have revised and added to the text to clarify our division of high and low flow environments. We state that because densities are low in the rhithron zone we had to combine samples with the transitional zone, and that there are other covarying variables other than flow that might affect shape of *T. areolatus*. These concepts are made clear in lines 103-108 and 116-123.

106-111 – Perhaps you can be more specific in terms of the breakdown of sizes? Bin them or tell the readers about the distribution? Reporting average size when you intentionally collected over a broad size range only tells the readers that the two groups have the same average size but nothing about the distribution of sizes.

Thank you for this suggestion. We added in size ranges for each of the locations for both the head and lateral analyses. See lines 131-137.

111 – Can you provide details to the level that the method can be replicated? The resolution of photos makes a difference in the precision/accuracy of your morphometric analysis. Camera and lens make/model, etc.

We added in additional information about which camera and lens make/model we used. See lines 127-128

122 – Is this the proper term for the rostral-most point on T. areolatus? Is the point at the tip of the premaxilla? Might try to be more specific here.

We changed the wording from “anterior tip of snout” to “rostral-most point.” See line 152.

123 –Can you be more specific with Point 5 in terms of the importance of this point, why you placed it here and what it tells the reader?

We use this landmark in conjunction with landmark 4 to estimate depth of the head. We do not feel it is necessary to provide additional information in the text about what this landmark captures. If we explain the purpose of this one landmark, we feel it would be necessary to provide descriptions for every single landmarks. See lines 154-155.

135 – Can you be more specific about point 2? Is this the occipital crest? Remember that these points must be homologous for proper landmarking.

This point represents the midline of the head at the back of the head. It is positioned on the midline as projected from point number 7. We modified the description of point two in the text. See lines ###

136-137 – It is unclear what point 6 means and how it is repeatedly placed. Is this the lateral-most boundary of the head at the level of the eye (i.e., the widest part of the head)? Perhaps you could say “lateral extent of the head indicated by the intersection of a line drawn perpendicular to points 1 & 2 that passes through the eye and the lateral aspect of the head along that line.” Or something more explicit.

This point is used to measure the width of the head at the eye. We modified the description of point 6 in the text. See lines 166-167.

162 – “12 rows (body) or 9 (head) rows” Consider being consistent about where parentheticals are placed in this sentence. It is helpful to the reader.

We updated the sentence to “12 rows (body) or 9 rows (head) to keep the parentheticals consistent. Thank you for catching this inconsistency. See line 196.

187-188 – Your explanation of Index methodology (above) is helpful in understanding how the interaction was developed. However, the way the topic sentence here is written is confusing/misleading. You state body shape differed significantly by flow and centroid size – but that is not what you report. I suggest you be explicit in how you open up the major finding of your results by stating exactly what was different, which is that body shape differs based on centroid size and between the three-way interaction of flow, centroid and index – this is different than saying that body shape differed sig by flow – which is what the reader takes away from this topic sentence, but is not necessarily true.

We have revised this sentence to more clearly state the statistical result of significance for all two-way and the three-way interactions. See lines 241-244.

193-194 – Is ontogeny driving the larger patterns you are seeing, particularly with your index interaction? How can you tease apart the effect of flow within this? I’m afraid that the significance of your index and all interactions using that term are driven by the overarching effect of centroid size. Clarifying how you can separate out these effects would be helpful to readers.

To be clear, the significant three-way interactions from our multivariate mixed model analysis (for both head and body) indicate that it is not just ontogeny or flow environment, but an interaction between the two. To make this clearer to the reader, we have added two new analyses to our paper. First, we have conducted a phenotypic change vector analysis that specifically tests for differences in magnitude and direction of multivariate shape change between flow environments over ontogeny. This analysis shows that in the body view ontogenetic shape change vectors differ significantly between low and high flow environments in magnitude, but not in direction. In the head view, ontogenetic shape change vectors differ significantly between low and high flow environments in direction, but not in magnitude. See lines 217-228 for an explanation of the methods of analysis and see lines 247-250 and 275-279 for the results of the analysis. The second analysis is a calculation of a divergence vector that allows us to visualize the maximum divergence due to flow environment at small and large sizes. We provide new thin-plate spline visualizations of these divergence patterns for comparison among groups. See figures 4 and 6 and lines 229-238 for an explanation of the methods of analysis and see lines 250-254 and 279-283 and for the results of the analysis. With the help of these two new analyses, we think the nature of this interesting interaction is made clear.

198 – You report the magnitude of shape change is significantly different between flow regimes. Be sure to cite the statistical results supporting this comment.

We have included a phenotypic change vector analysis to test for statistical significance in our three-way interaction. See lines 247-250.

210-211 – Same comment as line 187-188. The way you word this sentence is confusing and possibly misleading.

We have revised this sentence to more clearly state the statistical result of significance for all two-way and the three-way interactions. See lines 270-273

240 – The reporting of eye size is not obvious within Results. Is this true that adults have relatively larger eyes? I don’t see that in the warps and it doesn’t make sense with the allometry of eye size among other fishes. Eye size is strongly negatively allometric across ontogeny among fishes. Please check this reporting in your manuscript. Perhaps high flow adults have relatively larger eyes than low flow adults, but the way it is worded here suggests that eye size scales with positive allometry among high flow populations, and this should be clarified and expanded upon, if true.

Saying that adults had larger eyes was a mistake. Our updated figures represent the effect of ontogeny in each flow environment across all relative warps. We changed the results and discussion so that it is clear that change in eye size is part of the interaction between ontogeny and flow environment. See lines 325-330.

241-242 – Be sure to include the proper statistics in reference to this comment. Additionally, does this mean that high flow fish retain juvenile features into adulthood? Could you comment on this from a heterochrony/paedomorphic standpoint? Perhaps this is how the environments are mediating the ontogenetic trajectory of these fish – by maintaining juvenile-like characteristics among the high flow populations.

We have included a phenotypic change vector analysis to test for statistical significance in our three-way interaction. See lines 275-279. We have commented on potential reasons for differences in shape between flow environments over ontogeny. Since this is a descriptive study and we are only commenting on the shape of these organism, we do not feel comfortable calling this a paedomorphic change. Identification of paedomorphism/heterochrony requires a more detailed sampling of patterns of shape variation associated with reproductive maturity.

250-251 – It looks like the dorsal view of the head for warp 2 shows a wider aspect of the head at the mouth. I do not disagree that this could still be adaptive for the high flow environment, but I would argue that it shows a more fusiform shape. You will need to be more explicit here and in the results on what the shape changes between environments really are.

We have completely revised how we present our results. We have run two additional analyses outlined above and have been careful to integrate our explanation of body and head shape. See lines 250-254 and 279-283.

259 – Check this report of adults with relatively smaller eyes with what you report previously

Thanks for this comment. Adults do in fact have relatively smaller eyes compared to juveniles.

265-267 – There is no citation for the comment on apparent lack of reproductive barriers to gene flow and isolation of populations. Is this personal observation?

We have modified the text to indicate that there are no obvious barriers. See lines 341-344.

269 – You might consider stating at the end of the sentence something along the lines that a common garden experiment would have to be performed to directly address this inference.

We added an additional paragraph that discusses how a carefully designed, common-garden experiment would be useful in further addressing our inference. See lines 331-341 and lines 346-348

Figure 3 – It is difficult to see much difference among the warp line drawings. Consider a figure where they are made larger for the reader to see what is going on. Also, it is tough to interpret the data points on the graph. The data points are confined to as small section of the total space of the plot. Finally, low flow and high flow fish fell along perfectly flat, parallel lines? Perhaps zooming in could give the reader better resolution of the data points.

We generated new thin plate spline deformations from our new analyses. We made the thin plate spline images much larger so that the reader can easily see the morphometric changes that occur between environments and across ontogeny. See figures 4 and 6.

Reviewer #2: In this study the authors access body and head shape divergence of Trichomycterus areolatus between high and low flow river zones, comparing this change over a size range. They find both body and head shape is strong affected by size and, to a lesser extent, habitat zone. Interestingly, they show that the difference in head shape between high and low flow zones switches along size. Overall, I think the question and results are interesting and worth publishing. However, I think there is room for improving this manuscript. I find the introduction and discussion fairly narrow and does not set this study in a boarder context, especially for a journal like PLOS one that has a wide readership.

We have made major revisions to the introduction and discussion to broaden the scope of the manuscript. We have illustrated how morphometric traits can be selected for by the environment across many taxa, in addition to fish. See lines 38-51 and 298-307.

In addition, the authors do not mention the many confounding factors that could influence body and head shape in this study design.

We clearly state that other confounding factors could be present in the environment and could affect shape changes seen in *T.* areolatus. We have included explanations both in the methods and the discussion in our manuscript. See lines 104-108 and 331-340.

Finally, the clarity of the methods could also be improved.

To be clear, the significant three-way interactions from our multivariate mixed model analysis (for both head and body) indicate that it is not just ontogeny or flow environment, but an interaction between the two. To make this clearer to the reader, we have added two new analyses to our paper. First, we have conducted a phenotypic change vector analysis that specifically tests for differences in magnitude and direction of multivariate shape change between flow environments over ontogeny. This analysis shows that in the body view ontogenetic shape change vectors differ significantly between low and high flow environments in magnitude, but not in direction. In the head view, ontogenetic shape change vectors differ significantly between low and high flow environments in direction, but not in magnitude. See lines 217-228 for an explanation of the methods of analysis and see lines 247-250 and 275-279 for the results of the analysis. The second analysis is a calculation of a divergence vector that allows us to visualize the maximum divergence due to flow environment at small and large sizes. We provide new thin-plate spline visualizations of these divergence patterns for comparison among groups. See figures 4 and 6 and lines 229-238 for an explanation of the methods of analysis and see lines 250-254 and 279-283 and for the results of the analysis. With the help of these two new analyses, we think the nature of this interesting interaction is made clear.

Specific comments:

Line 42: I would not use “swimming style” and use “swimming performance” instead. Swimming style suggests the fish change swimming form, such as anguilliform to carangiform.

The introduction has been significantly revised and we no longer use this phrase in the text.

Line 47: Remove “within species (i.e. intraspecific variation)”. There is not need to define intraspecific. It is a commonly used term.

The introduction has been significantly revised and we no longer use this phrase in the text.

Line 60: Again, why have “(i.e. juveniles)”? Just use juveniles in place of small individuals.

We removed “(i.e. juveniles)” as suggested.

Line 74-82: Any information about the microhabitat use of Trichomycterus areolatus would be helpful here. Does it occur in habitats characterized by sand or wood structure; runs, riffles, or pools? For example, if this species only resides in pools habitat with high structure, any difference in river discharge may not influence this species since it would only reside in low flow.

Thank you for this suggestion. *Trichomycterus areolatus* does in fact occur in runs and riffles where there is rapid water velocity. We have added additional information on the microhabitat use of *T. areolatus* in the text. See lines 85-86.

Methods: This study compare individuals from sites with very different abiotic and biotic environments. This study design raises the concern that flow may not be the cause of any intraspecific shape differentiation between the zones, yet little is discussed on this topic.

Our revised manuscript clearly states that other confounding factors could be present in the environment and could affect shape changes seen in *T. areolatus*. We have included explanations both in the methods and the discussion in our manuscript. See lines 104-108 and 331-340.

Lines 92-94: Is a reason for combining the two zones into a high-flow grouping? Why not analyze all three separately? I would expect the high flow and low flow zone to have the greatest separation, with some overlap with the intermediate zone.

We have revised and added to the text to clarify our division of high and low flow environments. We state that because densities are low in the rhithron zone we had to combine samples with the transitional zone, and that there are other covarying variables other than flow that might affect shape of *T. areolatus*. These concepts are made clear in lines 103-108 and 116-123.

Lines 105-106: Why not use all the individuals?

Thank you for asking for clarification on this sentence. “Randomly selected” does not provide an accurate description of how we reached our final number of specimens collected. In fact, we used all of the specimens that were available to us unless they were preserved poorly. We have made updates in the text that clearly indicates how we selected the individuals that we used. See lines 128-129.

Line 143: A reader not familiar with geometric morphometrics may need to know what non-shape variation is being removed: position, orientation, and scale.

We added in that non-shape variation includes position, orientation and scale in the text for readers unfamiliar with geometric morphometrics. See lines 174-175.

Line 148-150: Why choose 12 and 9 axes? Is this based on a broken stick model, amount of variance explained, or some other method? This seem arbitrary and the figures in the manuscript only show 2 of the axes, seemly ignoring the rest that were included in the LMM.

We have justified the use of a subset of relative warps as those that accounted for more than 1% of total shape variation, and we have cited papers that use this same selection criterion. See lines 180-182.We also included an analysis that generates thin-plate spline deformations across all relative warps. See figures 4 and 6 and lines 229-238.

Lines 160-163: This should be explained in more detail. I was confused by this section. I believe the authors created a vector (single column) that included all relative warp (RW) axes (12 or 9 rows) for each specimen so that: row 1 is RW1 for specimen 1, row 2 is RW2 for specimen 1, and so on. In this case, the dependent variable for the multivariate linear mixed model is a vector (single column) and not a matrix (multiple columns)?

We added additional detail to the explanation of vectorization to specifically indicate that the response variable is transposed from a row in the matrix to a vector column of responses. See lines 197-198. We believe this makes the vectorization process clearer and we appreciate the suggestion.

Lines 188-193: Where does this information come from? I am assuming Figure 1.

Since we used two new analyses and generated new figures, the results have been significantly modified. We now provide specific statistical tests and visualizations of the effects of flow environment on ontogenetic shape change. See lines 241-254 and See figures 3 and 4.

Lines 195-199: The figure shows a slight difference between low and high flow which is expected (higher flow having narrower bodies and heads) but the confidence intervals overlap a fair amount for relative warp 2. This applies to the magnitude of shape change too. Can you say these are significantly different? At least one of your body shape axes are different but that is likely relative warp 1 and maybe not relative warp 2.

To be clear, our analysis is a multivariate mixed model, such that all selected relative warps are used simultaneously in the analysis. Thus, the interactions are based on the summed effect across multiple relative warps. To give an indication of how relative warps contributed to differences among groups, we included a new figure that plots least squares means for each relative warp for each size by flow environment combination. See lines 213-216 and Figures 3 and 5. We now explain in the results section how each relative warp is related to the overall shape change documented by the significant interaction terms. See lines 244-247 and 273-275.

Head shape shows clear patterns of separation. Interesting that larger fish had smaller heads in the body shape analysis but broader heads in the head shape analysis, being more compressed.

We have included new thin plate spline deformations to clearly illustrate the shape changes we see across all relative warps. See figures 4 and 6. These figures show that adults do in fact have shorter heads relative to the body that are broader. See lines 250-254 and 281-283.

Lines 257-258: Please expand why this pattern may be associated with the benthic nature or habitat use changes.

We have significantly modified the discussion. We added several sentences expanding how this pattern may be associated with the benthic nature of *T. areolatus*. See lines 308-324.
