# Supplementary material for: Ontogenetic shape trajectory of Trichomycterus areolatus varies in response to water velocity environment
Source: PLoS One. 2021 Jun 11;16(6):e0252780. doi: 10.1371/journal.pone.0252780 (PMC8195363; doi:10.1371/journal.pone.0252780)
Supplement: Attachment — Submitted filename: Response to Reviewers.docx [file pone.0252780.s003.docx]

Review: Ontogenetic shape trajectory of Trichomycterus areolatus varies in response to stream gradient

Re-Review:

The revision by Searle et al. is improved from the first submission, but the writing needs clarification throughout the manuscript. I have detailed line-by-line locations in the manuscript where the authors can consider clarifying, bolstering or specifying their arguments.

I still have a major issue with the take-home message being that stream gradient is the major feature driving the ontogenetic trajectories between the two groups. This is because your metric for quantifying stream gradient is the degree of river slope, which is 0.14 in the low-flow and 0.4/0.2 in the “high flow.” I understand that you grouped transitional flow with high so that you could have sufficient datasets for comparison, but I am not convinced this was the right way to go. Why combine transition flow data with high flow when the river slope in the transition is 0.2 degrees? This is closer to 0.14 of the low flow. Can you provide other data for flow in the low vs. high regimes, such as flow rate or flow volume? Your argument is that stream gradient is driving different ontogenetic trajectories in this species of fish. This is a super cool take-home message, so it needs to be clear that this is indeed the case.

We have thought through this argument and have gone back to a previous paper that detailed the variation among stream zones and our sampling locations. In the end, we agree with the reviewer that using gradient as our selective effect may not be the right way to present these differences. Our original intent was to represent variation in stream velocity, and we had used gradient as a surrogate, but further research suggested that gradient does not represent stream velocity very well in this system. Rather, we have now gone back and detailed the relevant differences between stream zones (see addition of Table 1) and have characterized the main difference as stream velocity itself. As it turns out, in this system, stream velocity is most different between the transitional and potamal zones, and stream velocity is the variable that many studies have used to characterize selective differences among stream environments.

To answer the question, we have rewritten major sections of the introduction and methods to make clear that stream velocity is the main variable we use to characterize differences in environment. We acknowledge that in this sort of study there are likely multiple, covarying variables that may contribute to differences in shape between environments. We cannot differentiate among these variables in our sampling design (because they do covary), so instead we have characterized the environmental differences as “water velocity environments” to include the general environment that is created by high-water velocity. This same reasoning strategy has been used successfully in other studies that rely on “natural experiments” or sampling of variation across multiple environments. Studies on differences in “predator environments” or “competitor environments” acknowledge that although predators (for example) are likely to have a dominant effect in trait selection, other covarying variables may contribute to or oppose such selection. We rely on the same argument in this paper – although water velocity has been shown to influence shape of fishes, other variables may exert some selective influence as well.

We make these arguments in paragraph 3 in the introduction, we are more specific about them in paragraph 1 of the methods (study site characterization), and we discuss the implications in paragraph 4 of the discussion. As you can see, we have made major revisions to the paper to focus on water velocity environments as the relevant form of environmental variation. We appreciate the reviewer’s insights on this point, and we believe that the paper now makes a strong argument for differences in shape between selective environments characterized as high-velocity environments or low-velocity environments.

Somewhat related, we have also addressed the issue by running the overall analysis with and without the few individuals from the rithron. We have demonstrated that the results we show are not dependent on the samples from the rithron. The statistical results and the estimates of the lsmeans are nearly identical, so we report the analysis from the full dataset (see the second paragraph in the methods).

The second major issue is in the interpretation or importance/novelty of the findings that ontogenetic trajectory is mediated by environment. The statistics indicate that this is the case (through the interaction), but there is no further clarity or understanding of what this means in terms of HOW ontogeny is redirected in a low- vs. high-flow environment. What happens to the shape of these fish during development? I understand that high-flow are more fusiform with longer heads, but you don’t need ontogenetic data to show this. What is new from this study is that you can detail what specific shape changes occur throughout ontogeny between the two environments. You might consider bolstering this argument.

To answer this concern, we have now done a better job of describing the specific ontogenetic trajectories that occur in each of the water velocity environments. We describe these trajectories as differing in both beginning shape (at small sizes) and ending shape (at large sizes). We then describe how the trajectories differ in multivariate magnitude and direction of shape change. Whereas, before we just gave the statistical outcome of the trajectory tests, we now give a description of each trajectory. We describe how shape change over ontogeny in the high-velocity environment is constrained compared to that in the low-velocity environment. These descriptions add substantially to the results, Furthermore, we now discuss these two characteristics of ontogenetic shape change in separate paragraphs in the discussion. Paragraphs 2 and 3 in the discussion compare, contrast, and explore the patterns of shape change we observe. We compare these patterns to other similar studies to put them in context.

I totally understand that it is deflating to receive a less-than-laudatory review. Your paper is getting there, but it needs (1) clarity in the writing, (2) further interpretation on how environment mediates ontogenetic trajectory (this is the novelty of your dataset) and (3) to reconcile the stream gradient issue (especially if this is the take-home message, i.e., in the title of your paper).

We appreciate the review, and it has led us to strive to clarify our writing to make the strength of this study apparent to readers. We have changed the focus of the environmental influence argument, and we have changed the title accordingly. We have further described and discussed how ontogenetic trajectories change between water velocity environments.

General comment not touched on below:

-	Wider heads and larger eyes in high flow could also have to do with prey type in high- flow environments. Prey type is a potential source of selection that could produce similar results.

The reorganization of the discussion no longer includes the elements mentioned above. However, we have added the idea that prey type is a potential source of selection to the introduction. See lines 58-60.

Line-specific comments:

27 – Sentence remains confusing (and it is important that this sentence is clear). Are you saying that head shape and body shape vary with respect to one another? This is kind of how this sentence reads.

Body and head shape can vary with respect to one another, but that is not the message we are trying to convey. We modified the sentence to indicate that body and head shape both vary in response to the water velocity environment over ontogeny. See lines 27-28

46 – This paragraph is on intraspecific variation in morphology, while the previous is on interspecific variation? I think you could clarify this for the reader. Also, you might include what the selective pressures were to cause these intraspecific morphological divergences in each example you provide.

We now use the words interspecific and intraspecific variation to make the main point of each paragraph clear to the reader. See lines 40-41 and 49-50. We added in additional information about the various habitats and selective pressures each study analyzed. See lines 50-54.

50 – Finally, for this paragraph, you might be careful with your wording in the last sentence. By suggesting that phenotypic variation can result from either natural selection or phenotypic plasticity, you are implying that plasticity is not under the control of natural selection, which I would argue is not true. Perhaps what you mean to say is that variation can have a genetic or plastic component? If this is true, you should clarify this language.

We modified the sentence as suggested to clarify the meaning. See lines 54-55.

52 – Your paragraph on ontogenetic shape change does not do justice to the vast literature on ontogenetic allometry among fishes, let alone vertebrates. From On Growth and Form (1917) to present day there are thousands of studies on the ontogeny of form. It would behoove you to give this subject more treatment both in the introduction and discussion of your paper.

We modified the paragraph in the introduction to include references to the many studies on ontogeny of form. See lines 70-73.

74 – “select for” or “result in/produce?” Select for implies a specific understanding of adaptive significance behind phenotypic change. In this case you are saying that environment would select for a particular ontogenetic strategy (i.e., fast growth rate or something). I think what you are trying to say is that environment can mediate ontogenetic trajectory to produce a different adult morphology from a similar juvenile starting morphology?

We now use different wording to clarify that environments influence ontogenetic trajectories. This is the basis of the interaction. See lines 93-95.

74 – 78 – It is unclear what is happening to male/female morphology between high and low predation environments. Are males the same throughout development or do they start different and undergo parallel shape change? Females start different but end up the same (converge)? This need clarification.

We clarified this sentence so that it states that the shape differences between predation environment in juvenile males is maintained in adult males; whereas, the shape differences between juvenile females is less pronounced in mature females. Females converge on a common shape as a consequence of pregnancy. See lines 84-90.

78 – 80 – Are you saying here that in this stickleback study the three environments produced three different morphologies? Or that marine differed from fresh? Please clarify. Also, is it that these studies did not look at shape change throughout development and that your study is doing this? It would be good for you to clarify exactly what has been done and where your study fits in.

In this study, three different ontogenetic trajectories were observed. Although two marine habitats were used, they differed from each other. See lines 90-92. Both of these studies did look at shape change throughout development. However, both of these studies did not specifically quantify the interaction between water velocity environment across ontogenetic trajectories in fishes. See lines 90-93.

80 – 82 – Consider deleting “in different environments over ontogeny” – this part of the sentence is repetitive.

We removed the phrase as suggested. See lines 93-95.

107 – Are you saying here that you grouped the environments into high-flow and low-flow and acknowledge that there are other features of these environments that might be causing the observed shape change that you quantify (other than flow)? You ultimately suggest (in the title) that shape change is a product of the stream gradient (which definitely implies flow), and not the confounding variables that also vary with environment.

I remain unconvinced of your reasoning to group the transitional zone with the high-flow. Your whole argument is in how flow speed shapes morphology, but the only values you present on flow speed are the slopes of these different zones (0.4, 0.2 and 0.14 degrees). You group the

0.4 and 0.2 together (lines 120-123) and compare this to the 0.14 and find difference in morphology and this is great, but I don’t know what to make of those differences in a functional way as a result of the “two different environments.” Can you provide a metric of flow velocity among the three sites, or flow volume (cubic feet per second) to better distinguish low- vs.

high-flow?

We have done as suggested here and as explained above this has resulted in substantial revisions to our arguments. See the more complete explanation above.

131 & 134 – Were these two totally different data sets for body shape and head shape? Or did you use mostly the same specimens, just one less for the head shape and 10 more for body shape? Also, were individuals tracked to compare head and body shape of the same individual? This would be important in the statistical analysis (i.e., repeated measures).

We used mostly the same specimens for both body and head shape. We have added an explanation as to how some individuals may be suitable for the head view but not the body and vice versa. See lines 222-223. We did not track individuals to compare joint shape change between body and head views, but the data are broadly overlapping. Each of the statistical analyses (head and body) were run independently, thus there is not statistical dependence here. The repeated measures analogy is based on multiple shape variables used in the analysis at one time, but we did not mix head and body relative warps in analyses. In the figures that show the shape variation associated with divergence vectors, one can see that patterns of shape change are consistent between body and head. For example, short heads relative to bodies in the low-velocity environment (body view) is the same as shorter heads overall in the head view in the same environment.

298 – I think I know what you are trying to say with this opening sentence, but it is awkward. It reads as if ontogeny is a selective pressure. Same with life history and environment. I believe that environment can provide selection, but I’m not sure that ontogeny and life histories are selective pressures so much as they are traits that can adapt as a result of selection. You should consider opening this section with a strong statement of your major result.

We have revised this confusing sentence. We now emphasize that environment is the selective force we are interested in analyzing across ontogenetic trajectories. See lines 347-348.

306 – You might consider being more specific here with what your results can say about how environment shapes ontogeny. You do not discern between genetic basis (that these are distinct populations that have evolved different developmental programs) and plasticity (that these populations are responding to their environment). It would take another study to tease these effects apart, but it could be worth acknowledging or specifying here what your data are capable of saying.

We have now specifically described how ontogenetic shape trajectories vary between water velocity environments. We have detailed this above. In addition, we have been clearer that we cannot tell whether these changes have a genetic or plastic basis, or both, and that it would take a common-garden environment experiment to sort this out (as well as the specific effect of each environmental variable associated with water velocity.

308 – What is your metric for fusiform shape? How was this calculated? The only other mention of “fusiform” is in the introduction section so it is hard for the reader to determine how you calculated this variable.

We used fusiform as a general descriptor to indicate narrower relative to more robust bodies. We have revised this sentence to use these more qualitative terms. See lines 388-390.

312 – Consider a new paragraph when transitioning to juveniles. This is a massive paragraph otherwise.

The discussion paragraphs have been substantially revised and restructured. This paragraph has been split up accordingly. See lines 397-407.

321 – Is “endorheic” the correct term here (closed drainage basin)? Does your study river terminate into a lake/basin? For those unfamiliar this might be worth clarifying. Also, you are talking about high flow environments in the preceding sentence, so a clearer transition is needed. 327 – Negative allometry in eye size (relatively large eyes in juveniles) is observed in most ontogenetic studies of fish and may be a developmental constraint more than a product of adaptation.

Endorheic is not the correct term. Thank you for catching this, we are embarrassed by this mistake. We now use the term hyporheic which was our original meaning. See lines 404-406. We modified the sentence so that the transition from high-velocity environment to benthic environments is clearer. See lines 402-406. The reorganization of the discussion no longer includes the elements mentioned above. However, we agree that negative allometry in eye size is likely a developmental constraint.

332 – 335 – Please clarify what you are saying here. Is the high flow environment punctuated by open basins of little-to-no flow? Are you saying that the benthic substrate of these environments is different? Please be more specific and clarify your point.

We now do a better job of characterizing environmental differences between sampling sites. See explanation above.

342 – 346 – This is the first mention that there are no reproductive barriers to gene flow within your study river system. This should come much earlier, or if you do mention it earlier, should be emphasized, because it is important to your findings, specifically that morphological differences might not have a distinct genetic basis and therefore would be the result of phenotypic plasticity.

We now discuss the presence of lack of reproductive barriers in our Methods section, as well as the discussion. We agrees that this provides strong support for our conclusion that what we see is likely the result of phenotypic plasticity. See lines 115-117.

Thank you for the opportunity to respond to reviews. We have carefully addressed each of the suggestions, and we think the paper is much improved.

Sincerely,

Peter Searle, for all authors
