# Supplementary material for: Ontogenetic shape trajectory of Trichomycterus areolatus varies in response to water velocity environment
Source: PLoS One. 2021 Jun 11;16(6):e0252780. doi: 10.1371/journal.pone.0252780 (PMC8195363; doi:10.1371/journal.pone.0252780)
Supplement: Attachment — Submitted filename: Response to Reveiwers.docx [file pone.0252780.s004.docx]

Review: Ontogenetic shape trajectory of Trichomycterus areolatus varies in response to water velocity environment

Reviewer #1: The authors addressed all concerns and the manuscript is easy to interpret and the take home message is clear. Two minor points:

1) do you have flow data on maximum velocity for the three different sites? I wonder if this would be interesting to include because it could be that certain flooding events (high flow) could be a major selective pressure, as opposed to average velocity (though the data suggest average velocity is enough to distinguish among the groups).

We agree that maximum velocity would be interesting to evaluate. However, we do not have a continuous measure that could capture this, because velocities were taken as point measures when we were in the field. In addition, we agree that average velocity distinguishes among the groups fairly well.

2) For the discussion, you might consider starting with your second paragraph and largely deleting the first paragraph. Perhaps you can use some of the language in the first paragraph to fill in what's missing if you start with the second, but the second is where the teeth of your results are reiterated and this makes for a stronger opening to the discussion.

We agree that our first paragraph in the discussion is largely not needed. We made the second paragraph our lead paragraph. We included a modified sentence from our first paragraph as the beginning sentence for our second paragraph (See line 347-348). This resulted in the removal of two citations (88 and 89). These citations were not critical citations.

Otherwise, I have no major comments and am grateful for your persistence and time spent improving the manuscript. It is great!

We appreciate the insightful comments provided during the revision of this manuscript. We are grateful for your patience as we worked through your suggestions. Thank you for helping us significantly improve our manuscript.

Thank you for the opportunity to respond to reviews. We have carefully addressed each of the suggestions, and we think the paper is much improved.

Sincerely,

Peter Searle, for all authors
